# Ascorbate Alleviates Fe Deficiency-Induced Stress in Cotton (*Gossypium hirsutum*) by Modulating ABA Levels

**DOI:** 10.3389/fpls.2016.01997

**Published:** 2017-01-04

**Authors:** Kai Guo, Lili Tu, Pengcheng Wang, Xueqiong Du, Shue Ye, Ming Luo, Xianlong Zhang

**Affiliations:** ^1^National Key Laboratory of Crop Genetic Improvement, Huazhong Agricultural UniversityWuhan, China; ^2^Key Laboratory of Biotechnology and Crop Quality Improvement of Ministry of Agriculture, Biotechnology Research Center, Southwest UniversityChongqing, China

**Keywords:** abscisic acid, ascorbate, cotton fiber, cytosolic APX, Fe deficiency

## Abstract

Fe deficiency causes significant losses to crop productivity and quality. To understand better the mechanisms of plant responses to Fe deficiency, we used an *in vitro* cotton ovule culture system. We found that Fe deficiency suppressed the development of ovules and fibers, and led to tissue browning. RNA-seq analysis showed that the *myo*-inositol and galacturonic acid pathways were activated and cytosolic *APX* (*ascorbate peroxidase*) was suppressed in Fe-deficient treated fibers, which increased ASC (ascorbate) concentrations to prevent tissue browning. Suppression of cytosolic *APX* by RNAi in cotton increased ASC contents and delayed tissue browning by maintaining ferric reduction activity under Fe-deficient conditions. Meanwhile, *APX* RNAi line also exhibited the activation of expression of *iron-regulated transporter* (*IRT1*) and *ferric reductase–oxidase2* (*FRO2*) to adapt to Fe deficiency. Abscisic acid (ABA) levels were significantly decreased in Fe-deficient treated ovules and fibers, while the upregulated expression of ABA biosynthesis genes and suppression of ABA degradation genes in Fe-deficient ovules slowed down the decreased of ABA in cytosolic *APX* suppressed lines to delay the tissue browning. Moreover, the application of ABA in Fe-deficient medium suppressed the development of tissue browning and completely restored the ferric reduction activity. In addition, ABA 8′-hydroxylase gene (*GhABAH1*) overexpressed cotton has a decreased level of ABA and shows more sensitivity to Fe deficiency. Based on the results, we speculate that ASC could improve the tolerance to Fe deficiency through activating Fe uptake and maintaining ABA levels in cotton ovules and fibers, which in turn reduces symptom formation.

## Introduction

With the increasing shortage of global available resources, efficient and sustainable agricultural production represents a major challenge (Schenkel, 2010; Gregory and George, 2011). Mineral nutrients must be of sufficient availability to ensure efficient crop productivity and product quality (Maathuis and Diatloff, 2013; Briat et al., 2015). Fe plays an important role in cellular function, and represents one of the essential micro-elements in plants and animals. Plants use Fe to ensure electron flow through the PSII-PSI complex during photosynthesis (Briat et al., 2007, 2015; Balk and Schaedler, 2014), and Fe plays a critical role in plant development because of its activity in regulating redox status. It is an indispensable cofactor in the respiratory chain and enzymes of tricarboxylic acid cycle, nitrate metabolism, biosynthesis of gibberellins and ethylene, amongst others (Lingam et al., 2011; Balk and Schaedler, 2014; Briat et al., 2015). Improvement in Fe utilization or enhancement of tolerance to Fe deficiency are important agronomic objectives.

Fe(III), is the most abundant type of iron in soil. However, due to the poor solubility of Fe(III) ions under aerobic conditions, especially in high pH and calcareous soils, plants usually display a shortage of Fe (Curie and Briat, 2003; Kobayashi and Nishizawa, 2012). In order to adapt to Fe deficiency stress, plants have evolved two strategies to take up Fe(III) from the soil, strategy I in non-graminaceous plants (including cotton, *Arabidopsis*, and pea) and strategy II in graminaceous (such as rice and maize and so on) (Kobayashi and Nishizawa, 2012; Thomine and Vert, 2013). Both strategies depend on the action of plasma membrane proteins in the cells of root epidermis (Brumbarova et al., 2015; Zelazny and Vert, 2015).

Strategy I plants absorb iron from the soil in a three-step process. Under Fe-deficient conditions, *Arabidopsis* roots secrete protons to the rhizosphere in a process dependent on the plasma membrane H^+^-ATPase AtAHA2 expressed in root epidermis cells, which increases the solubility of Fe(III) ions (Mlodzinska et al., 2015). Recently, the phenolics eﬄux zero (PEZ) protein was found to export phenolic compounds that were demonstrated to solubilize Fe(III) (Jin et al., 2007; Fourcroy et al., 2016). Once solubilized, Fe(III) entered the apoplast of root cells, and was chelated and reduced to Fe(II) by the ferric reductase–oxidase (FRO) family protein FRO2. This reduction has been proposed to be the rate-limiting step in Fe uptake (Robinson et al., 1999; Connolly et al., 2003). Reduced Fe(II) ions are subsequently transported into the root cell by iron-regulated transporter (IRT1) (Vert et al., 2002).

Transcription factors have been identified that are involved in the regulation of Fe absorption in strategy I plants, including bHLH transcription factors FIT1 (fer-like iron deficiency induced transcription factor), bHLH38/39/100/101, PYE (POPEYE), and MYB10 and MYB72 (Ling et al., 2002; Colangelo and Guerinot, 2004; Van der Ent et al., 2008; Long et al., 2010; Lingam et al., 2011; Wu et al., 2012; Brumbarova et al., 2015; Zhang et al., 2015). It has also been well-established that phytohormones participate in the Fe deficiency response, such as auxin, ethylene, and NO control Fe uptake by positively regulating the transcriptional activity of the *FIT1* gene (Chen et al., 2010; Lingam et al., 2011). The hormone abscisic acid (ABA) has been suggested to alleviate iron deficiency in *Arabidopsis* by promoting root iron reutilization and transport from root to shoot (Lei et al., 2014). ABA has been shown to involve in many aspect of plant growth and development and responses to an abundant of environmental stresses such as drought, salinity, cold, pathogen attack and UV radiation (Nambara and Marion-Poll, 2005; Cutler et al., 2010; Peleg and Blumwald, 2011; Finkelstein, 2013). It also proved that ABA participated in the Fe metabolism and ferritin synthesis. For the exogenous application of ABA could induce the accumulation of ferritin mRNA (Lobreaux et al., 1993). A set of ABA and osmotic stress response genes are co-regulated by Fe signaling or by the gene *CPL1-2* (*RNA polymerase II C-terminal domain-phosphatase-like1*) which act upstream of FIT, negatively affecting the mRNA availability of *FIT1*, *FRO2* and *IRT1* in *Arabidopsis* (Aksoy et al., 2013). However, the molecular mechanism of ABA involved in Fe deficiency in plant is still unknown, even though it has been shown that ABI5 is involved in the ABA-mediated alleviation of chlorosis under Fe deficiency in *Arabidopsis* (Lei et al., 2014).

Recent evidence suggests the existence of a role for ascorbate (ASC) and glutathione (GSH) in adaption to Fe deficiency (Ramirez et al., 2013; Shanmugam et al., 2015). In animals, ASC is involved in iron accumulation by astrocytes, an important contributor to iron homoeostasis in the mammalian brain (Lane et al., 2010). In higher plants, ASC and GSH treatments can alleviate Fe deficiency-induced chlorosis and restore chlorophyll content in Fe-deficient *Arabidopsis* seedlings (Ramirez et al., 2013). Recently, a study indicated that the decrease in GSH concentrations in the GSH biosynthesis pathway mutant *zir1* significantly reduces plant tolerance to Fe deficiency and alkaline soils, while transgenic *ZIR1* overexpressors exhibit increases in GSH content and enhanced tolerance to Fe deficiency (Shanmugam et al., 2015). However, the underlying molecular basis for ASC or GSH effects on Fe deficiency, including links with plant hormone signaling systems, are not well-understood.

There are also evidences to suggest that ASC is a part of a Fe transport system to overcome the Fe deficiency in dicotyledonous embryos. It was found that the eﬄux of ASC in pea embryos could increase ferric reduction (Grillet et al., 2014). Similarly, the mutant of ascorbate biosynthesis genes in *Arabidopsis* (*vtc2-4*, *vtc5-1*, and *vtc5-2*), was significantly reduced the ferric reduction activity and Fe content in embryos (Grillet et al., 2014). In the mammalian brain, the acquisition of iron in astrocytes was dependent on the eﬄux of ASC (Lane et al., 2010). Extracellular ASC reduces non-transferrin-bound Fe to promote the uptake of Fe by K562 cells (Lane and Lawen, 2008). Based on the results, it proposed that ASC medicated ferric reduction step is critical for Fe uptake in the delivery to the final targets.

Cotton (*Gossypium hirsutum*) is a dicotyledonous plant that employs Strategy I for regulating uptake of Fe ions. Cotton fibers, produced on the ovule surface, are an important commercial crop product (Lee et al., 2007; Haigler et al., 2012). The *in vitro* ovule culture system has been developed as a valuable experimental system for the investigation of fiber differentiation, initiation and elongation (Beasley, 1973; Beasley and Ting, 1973), and is potentially useful for exploring the relationship between cell differentiation and mineral nutrients, including the effects of nutrient deficiency on plant cell biology.

In this study, we investigated the effects of transgenic (RNAi) suppression or overexpression of cytosolic *APX* on ASC levels and tolerance to Fe deficiency in cotton. We also investigated the link with ABA levels and ferric reduction activities. Our results provide evidences that ASC improves tolerance of Fe deficiency through effects on ABA signaling and activation of Fe uptake.

## Materials and Methods

### Materials

Cottons [*Gossypium hirsutum* YZ1, xuzhou142 and *xuzhou142 lintless-fuzzless* (*xu142-fl*)] were grown in experimental fields at Huazhong Agricultural University, Wuhan, Hubei province, China or in greenhouse conditions (28–35°C by day and 20–25°C by night with a 16/8 h light/dark cycle). Transgenic cotton lines, *GhAPX1* overexpressed lines OA15 and OA17, *GhAPX1* specific suppressed lines IAU20 and IAU22, cytosolic APX suppressed lines IAO24 and IAO167 and controls (Null and wild type cotton YZ1) are as described previously (Guo et al., 2016), and were grown in the field or the greenhouse prior to ovule culture.

### *In vitro* Ovule Culture

Ovule culture was performed essentially as previously described (Guo et al., 2016). On the afternoon of the day of flowering (0.5 DPA), flowers were collected, floral organs were peeled off, and the ovaries were surface sterilized with 0.1% HgCl_2_ for 15 min. Sterilized ovaries were washed with sterile distilled and deionized water (ddH_2_O) five times. After carpels of sterilized ovaries were peeled off, ovules were stripped off and 15–20 ovules per 50-mL flasks were suspended on BT medium (Beasley and Ting, 1973) and were cultured at 30°C in darkness in the presence of 0.5 μM GA_3_ and 5 μM IAA. The basic concentration of iron in the medium was 30 μM in the form of Fe(II)-EDTA. Six concentrations of Fe(II)-EDTA (0, 15, 30, 60, 120, and 240 μM) were used for experiments. To measure fiber length, cultured ovules were rinsed with ddH_2_O, and heated to 100°C until the fibers straighten. The length of the fiber was measured manually with a ruler after fibers were pulled softly on the filter paper into a straight line. Eight ovules were selected for the fiber length measurement in one bottle of cultured ovules. Three biological replicates were performed.

### Total RNA Extraction and Gene Expression Analysis

Fibers and ovules collected from ovule culture were ground into powder in liquid nitrogen. Total RNA extraction was performed according to the method described previously (Zhu et al., 2005). cDNA was synthesized with M-MLV Reverse Transcriptase (Promega, Madison, WI, USA) according to the manufacturer’s instructions. *GhUB7* (DQ116411) was used as the internal control to judge the quantity of the reverse transcription. Reverse transcription polymerase chain reaction (RT-PCR) was performed using an BIO-RAD MyCycler^TM^ thermal cycler (Bio-Rad, USA), and qRT-PCR was performed as previously described using an Applied Biosystems 7500 Real-Time PCR System (Guo et al., 2016). Primers used in the study are listed in the Supplementary Table 1.

### Screening of Differentially Expressed Genes (DEGs) by RNA-seq

0.5 DPA (days post anthesis) ovules of cotton YZ1 were cultured in medium containing 0 μM Fe (-Fe), 60 μM Fe (+Fe), and 240 μM Fe (++Fe). Each treatment contains two biological replicates, and each biological replicate had six technical repeats. After 10 days of culture, fibers were gently removed from ovules with a pestle in liquid nitrogen and ground into powder for total RNA extraction (Guo et al., 2016). Six samples were collected for RNA-seq by Illumina HiSeq^TM^2000 (BGI, Wuhan, China). The coding sequence (CDS) and genome sequences of *G. hirsutum* TM-1 (BioProject ID: PRJNA248163) were used as the reference for read mapping and gene annotation. A total of 12 M reads were obtained for each sample. Six samples were divided into three groups (-Fe, +Fe and ++Fe). Screening of differentially expressed genes (DEGs) between each pair of groups was carried out by pairwise comparison using the NOIseq method (Tarazona et al., 2011). The filtering parameters used were fold ≥ 2 and RPKM ≥ 1. RPKM (reads per kb per million reads) was used as a value to represent the expression level of gene, calculated as RPKM(A) = 10^6∗^C/(NL/10^3^) where C is the number of reads uniquely aligned to gene A; N is the total number of reads; L is the number of bases of gene A. GO term enrichment analysis of DGEs was carried out by Blast2GO software with Fisher’s Exact Test. PCA analysis of RNA-seq data in six simples was performed with Genesis software using RPKM values.

### Ionome Quantification with ICP–MS

Ovules of cotton YZ1 were cultured in three concentrations of Fe (0, 60, and 240 μM). After 10 days culture, fiber-bearing ovules were washed with ddH_2_O for three times. After the water was removed with filter paper, fiber-bearing ovules were frozen in the liquid nitrogen and ground into powder with a pestle. The powder was dried in a vacuum freeze drier (LABCONCO FreeZone^®^2.5, USA) to achieve constant weight for ionome quantification according to the method described previously (Yang et al., 2014). Samples of dry mass 0.2 g were digested with 65% nitric acid in a MARS6 microwave (CEM MARS 6, USA) at a temperature gradient of 120–180°C for 45 min. After the sample was completely digested, the nitric acid was removed by evaporation at 160°C for 40 min, and then diluted with ddH_2_O. The metal contents of the samples were determined by inductively coupled plasma-mass spectrometry (ICP-MS, Agilent 7700 series, USA).

### Assay of APX, CAT, and Ferric Reduction Activity

Fiber-bearing ovules were cultured in six concentrations of Fe (II) EDTA (0, 15, 30, 60, 120, and 240 μM) and used for the assay of APX and CAT enzyme activity according to the method described previously (Guo et al., 2016). Treated fiber-bearing ovules were washed with 5 mL of 0.5 mM CaSO4 and the water was removed with filter paper. Ferric reduction activity was assayed according to a previous method with a few modifications (Chen et al., 2010). Fresh samples (0.05–0.1 g) were placed into a test tube filled with 1 mL assay solution consisting of 0.5 mM CaSO4, 0.1 mM 4-morpho-lineethanesulfonic acid, 0.1 mM bathophenanthrolinedisulfonic acid disodium salt hydrate (BPDS, B1375, Sigma, USA), and 100 μM Fe(III)-EDTA at pH 5.5. After incubation in a dark room at 30°C for 30 min, with swirling at 10 min intervals, the absorbance of the assay solutions was measured at 530 nm using an Enspire^®^ Multimode Plate Reader (PerkinElmer, USA). A series of 1–16 μL of 10 mM Fe(II)-EDTA solution were added to a 1 mL assay solution to construct a standard curve, according to the absorption at 530 nm and the concentration of Fe(II)-BPDS.

### Hormones Quantification

Fe-deficient or -sufficient fiber-bearing ovules of YZ1 and *GhAPX* transgenic cottons were used for the quantification of endogenous hormones. Fibers were removed from the ovules in liquid nitrogen and ground to a powder. Samples (0.1 g) were extracted with 750 μL of cold extraction buffer (methanol: water: acetic acid, 80:19:1, v/v/v) supplemented with an internal standard (10 ng ml^-1^ of ^2^H-6-ABA, DH-JA, and H5-IAA) and shaken for 16 h at 4°C in the dark. Quantification of hormones was made by LC–GC/MS according to the method reported previously (Liu et al., 2012).

### Quantification of ASC, Glucose, Fructose, and Sucrose

Assay of ASC content was performed according to a previous method (Guo et al., 2016). For the assay of glucose, fructose, and sucrose in ovules and fibers treated with -Fe, +Fe and ++Fe, fibers were removed from the ovules in liquid nitrogen and ground to a powder. Samples (0.07 g) were extracted in a 10 mL tube with 2 mL 80% ethanol, heated to 80°C for 30 min, then centrifuged at 12,000 × *g* for 15 min. The supernatant was transferred to a new 10 mL tube. The precipitate was extracted with 80% ethanol two more times to produce a total 6 mL extraction for the assay of glucose, fructose, and sucrose as previously described (Zhao et al., 2010). Microplates, without standards added, were placed in an oven at 50°C for 30 min to remove ethanol and 20 μL of extraction was added to each well. Standards were 0 (blank), 0.005, 0.0125, 0.025, 0.050, 0.125, 0.250, or 0.500 mg mL^-1^ glucose in 20 μL of ddH_2_O. For glucose assays, 20 μL of ddH_2_O was added to each well-containing dried sample extract, and then a 100 μL mixture of the glucose assay reagent (Sigma, GAHK-20) was added to all wells (including blank and standards) in a microplate. After incubating at 30°C for 15 min, the absorbance of each well was measured at 340 nm using an Enspire^®^ Multimode Plate Reader (PerkinElmer, USA). Then, 10 μL of prepared Phosphoglucose Isomerase (2.5 EU/μL, P5381, Sigma, USA) was added to each well and after incubation for 15 min at 30°C, absorbance was measured at 340 nm for the assay of glucose and fructose contents. The last, 10 μL of the prepared invertase solution (165 EU/mL, I4504, Sigma, USA) was added to each well and absorbance was determined at 340 nm after incubating for 60 min at 30°C for the assay of sucrose content. The standard curve was constructed with the absorbance and the concentration of glucose standard.

## Results

### Fe Deficiency Induced Browning of Ovules and Fibers

**FIGURE 1:**
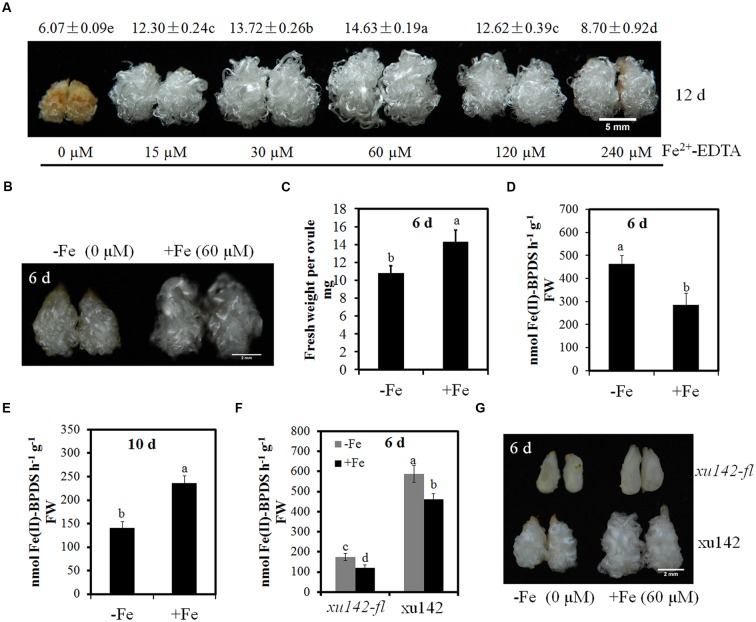
**The phenotype and ferric reduction activity of fiber-bearing ovules cultured with different concentrations of Fe. (A)** 0.5 DPA ovules of YZ1 were cultured for 12 days in medium with various concentrations of Fe (0, 15, 30, 60, 120, and 240 μM). Fe deficiency (0 μM) suppressed the development of ovule and fiber and resulted in tissue browning. Scale bar: 5 mm. Phenotype **(B)**, fresh weight per ovule **(C)** and ferric reduction activities **(D)** of YZ1 0.5-DPA-ovules cultured for 6 days under Fe deficient (-Fe, 0 μM Fe) or Fe sufficient (+Fe, 60 μM Fe) conditions. Scale bar: 2 mm **(B)**. **(E)** Ferric reduction activities of ovules cultured under Fe deficient or Fe sufficient condition for 10 days. Ferric reduction activities **(F)** and phenotype **(G)** of xuzhou142 (xu142) and xuzhou142 *fuzzless-lintless* (*xu142-fl*) mutant cultured under Fe deficient (-Fe, 0 μM Fe) or Fe sufficient (+Fe, 60 μM Fe) conditions for 6 days. Error bars represent SD. Every sample includes four biological replicates. Scale bar: 2 mm **(G)**. Values with different letters above bars in the histogram indicate significant differences (one-way ANOVA and Duncan’s multiple comparisons, *P* < 0.05).

Six levels of Fe were set up in ovule culture medium to investigate the effects of different Fe concentrations on ovule and fiber development. Fe deficiency (-Fe, 0 μM Fe) not only suppressed ovule and fiber development, but also led to tissue browning (**Figure 1A**). After 12 days culture, the fiber length on 60 μM of Fe (+Fe) was the longest compared with other treatments, and low levels (0 or 15 μM) or very high levels (240 μM) of Fe significantly inhibited ovule and fiber development (**Figure 1A**).

### The Change of Ferric Reduction Activity Was Coupled with the Ovule Fe Deficiency Response

To identify whether the fiber-bearing ovule shows the typical responses to Fe deficiency, we measured ferric reduction activity and fresh weight of fiber-bearing ovules after 6 or 10 days culture. After 6 days culture, ovules and fibers were significantly suppressed under Fe deficient condition (-Fe, 0 μM Fe) and fresh weight per ovule was significantly decreased compared with Fe sufficient condition (+Fe, 60 μM Fe) (**Figures 1B,C**). At 6 days no browning was occurred and ferric reduction activity of fiber-bearing ovules under Fe-deficient conditions was higher than in Fe-sufficient conditions (**Figure 1D**). By 10 days culture, ferric reduction activity decreased in Fe deficient cultures compared with Fe-sufficient ovules (**Figure 1E**). To exclude the possibility that changes in ferric reduction activity was linked to fiber growth, we measured the ferric reduction activity in variety xuzhou142 (xu142) and its mutant xuzhou142-*fuzzlesslintless* (*xu142-fl*) which has no fibers, under Fe-deficient and Fe-sufficient conditions (**Figures 1F,G**). Results showed that ferric reduction activities were both increased in xu142 and *xu142-fl* under Fe deficient condition after 6 days culture, which suggested that the change of ferric reduction activity was not caused by the fiber (**Figures 1F,G**). Therefore, Fe deficiency induces the increase of ferric reduction activity in the early growth period (6 days) which is co-occurred with maintenance of ovule and fiber development, but after a longer culture (10 days), the decreased ferric reduction activity is co-occurred with the browning of ovules and fibers.

### Oxidative and Biotic Stress Response were Enriched in Fe Deficiency-Fiber

**FIGURE 2:**
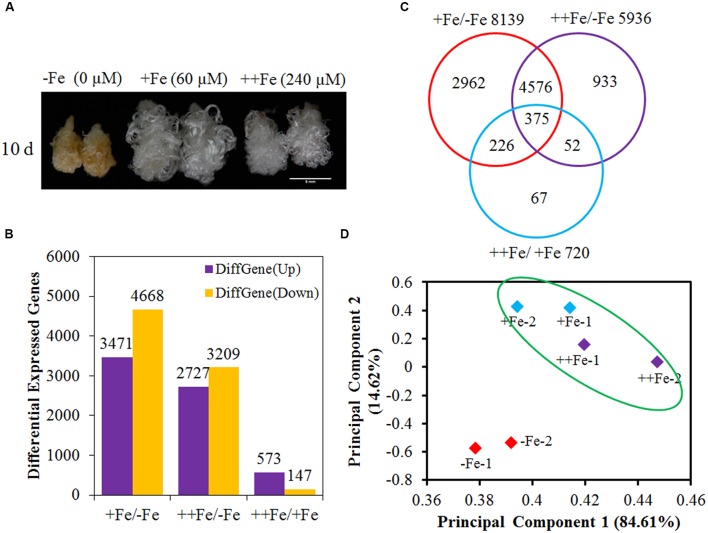
**Screening and analysis of differentially expressed genes (DEGs) in fibers cultured with three concentrations of Fe for 10 days. (A)** Phenotype of ovules and fibers cultured for 10 days in medium with three concentrations of Fe. Scale bar: 5 mm. -Fe, 0 μM Fe; +Fe, 60 μM Fe; ++Fe, 240 μM Fe. **(B)** DEGs in fibers between each two treatments with Fe. +Fe/-Fe indicates the gene expression levels in +Fe compared to the gene expression levels in -Fe. ++Fe/-Fe indicates the gene expression levels in ++Fe compared to the gene expression levels in -Fe. ++Fe/+Fe indicates the gene expression levels in ++Fe compared to the gene expression levels in +Fe. **(C)** Venn diagram of DEGs in the response to three levels of Fe. **(D)** PCA plots of six simples treated with three concentrations of Fe (each treatment has two biological repeats) based on RNA-seq data.

To gain insight into the regulatory networks responding to Fe deficiency, we compared gene expression levels using RNA-seq in fibers cultured in -Fe (0 μM), +Fe (60 μM), and ++Fe (240 μM) for 10 days (**Figure 2A**). We found that 8139 genes were differentially expressed (fold change ≥ 2, RPKM ≥ 1) in +Fe compared to -Fe (3471 up and 4668 down), and 5936 genes (2727 up and 3209 down) in ++Fe compared to -Fe. However, under excess Fe conditions (++Fe), 720 genes were significantly changed (573 up and 147 down) in the comparison of ++Fe vs. +Fe. Moreover, the expression levels of 4951 genes were simultaneously changed in the comparison of +Fe vs. -Fe and ++Fe vs. -Fe (**Figures 2B,C**). This indicates that +Fe- and ++Fe-treated fibers show a similar response to the Fe level. GO analysis shows that the 3471 upregulated genes in the comparison +Fe vs. -Fe are enriched for the functional categories *cytoskeleton and microtubule processes*, *GTPase activity*, *fatty acid biosynthesis and metabolic processes*, *organic acid biosynthesis processes* amongst others (Supplementary Table 2). The 4668 downregulated genes in the comparison of +Fe/-Fe are enriched for *oxidoreductase activity* and *nucleic acid or DNA* or *metal binding pathway* and *biotic stimulus response* (Supplementary Table 3). Results for ++Fe vs. -Fe were similar with those in for +Fe vs. -Fe (Supplementary Tables 4 and 5). Meanwhile, PCA analysis with RNA-seq data of six simples showed that Fe deficiency treated simples displayed a big distance to normal Fe or excess Fe treated simples, and normal Fe or excess Fe treated simples are clustered together (**Figure 2D**). These results suggest that Fe deficiency induces oxidative stress and biotic stress pathways or inhibites cytoskeletal and fatty acid biosynthetic processes, which may suppress ovule and fiber development.

### Fe Deficiency Cultured Fiber-Bearing Ovules Exhibited Typical Strategy I Fe Deficiency Response

**FIGURE 3:**
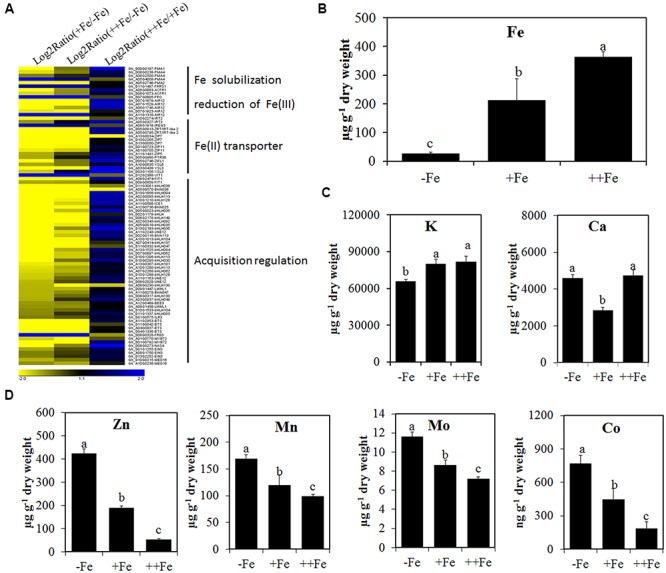
**The expression level changes of Fe deficiency response marker genes and the contents of ions in 10 days fibers or ovules treated with three concentrations of Fe. (A)** Cluster of marker genes responding to Fe deficiency (-Fe) based on the expression levels in 10 days fibers treated with three concentrations of Fe. -Fe, 0 μM Fe; +Fe, 60 μM Fe; ++Fe, 240 μM Fe. Contents of Fe **(B)**, K and Ca **(C)**, Zn, Mn, Mo, and Co **(D)** in ovules treated with three concentrations of Fe for 10 days. Each treatment includes three biological replicates. Error bars represent SD. Values with different letters above bars in the histogram indicate significant differences (one-way ANOVA and Duncan’s multiple comparisons, *P* < 0.05).

To further investigate the Fe deficiency response of fibers, we analyzed the expression of marker genes for iron uptake regulation and iron transport in 0, 60, and 240 μM Fe treatments. The genes were clustered into three classes and the majority of those genes were induced by Fe deficiency (**Figure 3A**). The first class included genes involved in the solubility of ferric ions and the reduction of Fe(III) to Fe(II), such as *plasma membrane atpase* (*PMA*) and *FRO* genes. The second class of genes included iron transporters, such as *IRT1/2*, *IREG3*, *ZIP*, *ZIFL1*, and *YSL*. The third class of genes included transcription factors regulating Fe uptake, such as the bHLH type of transcription factors *FIT1*, *bHLH38/92/103/113* and other regulators such as *ICE*, *FRD3 MYB72*, and *EIN3* (**Figure 3A**). It has been demonstrated that these marker genes are involved in Fe deficiency response or Fe uptake in *Arabidopsis* and tomato (Brumbarova et al., 2015). This shows that cotton ovule and fibers show a typical response to Fe deficiency.

It has been shown that Fe deficiency induces the accumulation of Zn, Mn, Mo, and Co in plants (Yang et al., 2010). We therefore examined the ionome in 10 days fiber-bearing ovules cultured in the presence of three concentrations of Fe. The contents of Fe and K were significantly reduced and Ca was increased in Fe-deficient ovules (**Figures 3B,C**). Fe deficiency also led to significant increases in contents of Zn, Mn, Mo, and Co (**Figure 3D**). These results suggest that the Strategy I Fe deficiency response occurs in Fe-deficient ovules and fibers.

### Fe Deficiency Suppresses the Expression and Enzyme Activity of Cytosolic *APX*

Due to its critical role in electron transfer during photosynthesis and respiration, Fe contributes to the production of reactive oxygen species (ROS) in plants (Briat et al., 2015). The expression levels of ROS related genes were clustered in -Fe, +Fe, and ++Fe treatments. ROS generation genes, such as *RBOHA/B/C/D* and *AOX1*, were significantly induced by Fe deficiency in 10 days fibers (Supplementary Figure 1; Supplementary Table 6). Of the ROS scavenging genes such as *CAT1*, *APX1/2*, *SOD*, *GLRX*, *FR*, *MT* and *BCP*, the majority were suppressed by Fe deficiency, and the majority of thioredoxin, cupredoxin, peroxidase, and glutathione *S*-transferase encoding genes were induced by Fe deficiency (Supplementary Figure 1; Supplementary Table 6). This suggests that Fe deficiency changes the redox status in fiber and ovule.

To confirm these results, RT-PCR and activity assays were carried out on the heme containing protein genes *APX* and *CAT*. The expression levels and enzyme activities were significantly suppressed by Fe deficiency in the fiber-bearing ovules at 10 days of culture (Supplementary Figure 2). We concluded that the redox status of ovule and fiber cell was disturbed under Fe deficiency condition.

### The Activation of Biosynthesis and Suppression of Cytosolic *APX* Leads to the Accumulation of ASC in Fe-Deficient Ovule and Fiber

**FIGURE 4:**
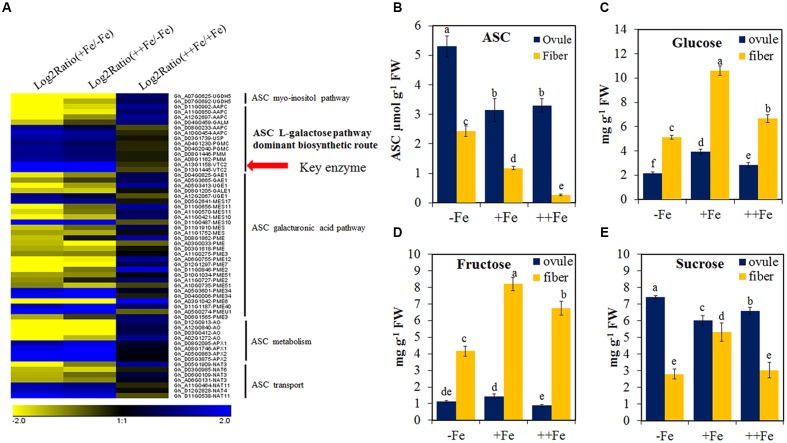
**Fe deficiency leads to an increase in ASC in ovules and fibers after 10 days culture. (A)** Cluster analysis of the expression levels for ASC synthesis and metabolism related genes responding to Fe deficiency. -Fe, 0 μM Fe; +Fe, 60 μM Fe; ++Fe, 240 μM Fe. **(B)** The contents of ASC increased in ovules and fibers treated with Fe deficiency for 10 days. Each treatment includes six biological replicates. Error bars represent SD. The concentrations of glucose **(C)**, fructose **(D)**, and sucrose **(E)** in ovules and fibers after treatment with three concentrations of Fe for 10 days. Each treatment includes six biological replicates. Error bars represent SD. Values with different letters above bars in the histogram indicate significant differences (one-way ANOVA and Duncan’s multiple comparisons, *P* < 0.05).

Fe deficiency suppressed most of the ROS scavenging enzyme activities and activated the expression of ROS generation genes, which led to the increasing visibility of the browning over 10 days until recognizable (**Figure 1**; Supplementary Figures 1 and 2). Therefore, some protection mechanism might exist under Fe deficiency condition. We considered that ASC might be one of the factors because of its anti-oxidant properties. The ASC biosynthetic and metabolic pathways and ASC contents were examined in ovules and fibers treated with three levels of Fe. Of the four known pathways of ASC biosynthesis, three were affected by the level of supplied Fe. Two pathways, the *myo*-inositol and galacturonic acid pathway, were activated by Fe deficiency. The mainly biosynthetic L-galactose pathway was suppressed by Fe deficiency (**Figure 4A**). In particular, the expression level of the rate limiting enzyme gene *VTC2* was significantly downregulated in Fe deficiency (**Figure 4A**).

The expression level of the ASC metabolic gene *ascorbate oxidase* (*AO*) was induced in Fe-deficient fiber, while cytosolic *APX* genes were inhibited by Fe deficiency (**Figure 4A**). However, the ASC contents in Fe-deficient cultured ovules and fibers increased (**Figure 4B**). The content of glucose, the raw material for ASC biosynthesis, and fructose significantly decreased under Fe-deficiency (**Figures 4C,D**). Although, the content of sucrose increased in ovules under Fe deficiency, it decreased in the fibers (**Figure 4E**). These results show that the activation of *myo*-inositol and galacturonic acid pathways and the suppression of cytosolic *APX* contribute to the accumulation of ASC in the Fe deficiency fiber-bearing ovules.

### Cytosolic *APX*-Silenced Cotton Shows a Higher Tolerance to Fe Deficiency

**FIGURE 5:**
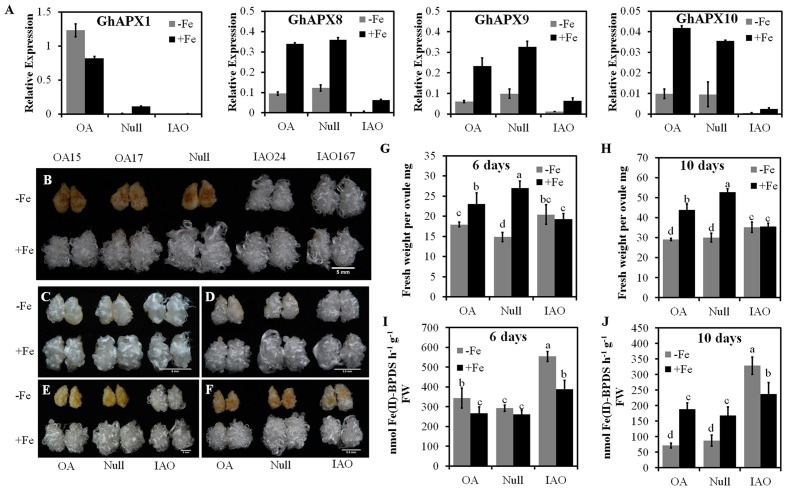
**Cytosolic *APX* RNAi lines (IAO) showed higher tolerance than the control (Null) to Fe deficiency stress. (A)** Quantitative reverse transcription polymerase chain reaction (qRT-PCR) analysis of cytosolic *APX* genes (*GhAPX1, GhAPX8, GhAPX9*, and *GhAPX10*) in ovules of transgenic cottons and the control cultured for 10 days under Fe-deficient or Fe-sufficient conditions. **(B)** Phenotype of *GhAPX1* overexpressor (OA15/OA17), the control (Null) and cytosolic *APXs* suppressed (IAO24/IAO167) fiber-bearing ovules treated for 10 days under Fe-deficient and Fe-sufficient conditions. Scale bar: 5 mm. The dynamic phenotypes of *GhAPX1* overexpressor (OA), control (Null) and cytosolic *APXs* suppressed (IAO) fiber-bearing ovules treated for 6 days **(C)**, 8 days **(D)**, 10 days **(E)** and 12 days **(F)** under Fe-deficient condition. Scale bar: 5 mm **(C–F)**. Fresh weight per ovule of transgenic cotton ovules cultured in Fe-deficient or -sufficient medium for 6 days **(G)** or 10 days **(H)**. Ferric reduction activities of transgenic cotton cultured for 6 days **(I)** or 10 days **(J)** under Fe-deficient or -sufficient conditions. -Fe, 0 μM Fe; +Fe, 60 μM Fe. Error bars represent SD. Every sample includes four biological replicates. Values with different letters above bars in the histogram indicate significant differences (one-way ANOVA and Duncan’s multiple comparisons, *P* < 0.05).

To investigate whether Fe deficiency-induced browning is linked to endogenous ASC content, we engineered ASC content in cotton through the modification of the expression level of cytosolic *APX*, by generating transgenic *GhAPX1* overexpressing lines (OA15/OA17) and cytosolic *APX* RNAi lines (IAO24/IAO167), for comparison with negative controls (Null) (Guo et al., 2016). Results in **Figure 5A** show that *GhAPX1* is upregulated in the ovules in overexpression lines, and four cytosolic *APX* genes are significantly suppressed in the RNAi lines. Four cytosolic *APX* genes were suppressed by Fe deficiency in the ovules cultured for 10 days, consistent with previous results (**Figures 4A** and **5A**; Supplementary Figure 1).

After 10 days culture under Fe deficient condition, overexpression lines OA15 and OA17 and Null showed the browning phenotype, and ovule and fibers were suppressed compared with them under Fe sufficient condition. In contrast, the RNAi lines IAO24 and IAO167 showed less sensitivity to Fe-deficient conditions. These ovules and fibers showed significantly less browning than Null tissues, and there was no inhibition of development of fiber-bearing ovules compared to Fe-sufficient conditions (**Figure 5B**).

To confirm the responses of the RNAi lines, time-course analyses of transgenic ovules and the control under Fe-deficient and -sufficient conditions were carried out from 6 to 12 days. After 6 or 8 days culture under Fe-deficient conditions, ovules of overexpressing, Null and RNAi lines showed no browning but the development of ovules and fibers in overexpressing and Null lines was suppressed compared to Fe-sufficient treatment (**Figures 5C,D**). However, ovules and fibers of the RNAi lines showed a similar response whether cultured under Fe-deficient or Fe-sufficient conditions, and fresh weight per ovule in the RNAi tissues did not decrease after 6 days culture under Fe deficiency (**Figure 5G**). After 10 days culture under Fe-deficient condition, overexpressor and Null lines showed significant browning of ovules and fibers, but RNAi tissue was still white at 10 days and only began to show browning at 12 days (**Figures 5E,F**), and the fresh weight per ovule was still similar to that under Fe-sufficient conditions (**Figure 5H**). Suppression of expression of cytosolic *APX* led to a higher tolerance of Fe deficiency than either overexpression or wild type levels of expression.

Ferric reduction activity is associated with tolerance to Fe deficiency (Connolly et al., 2003). We found that ferric reduction activity increased in Fe deficient ovules after 6 days of culture and ovule and fiber were white, but after 10 days culture, ferric reduction activity decreased and ovule and fiber were brown (**Figures 1** and **5I,J**). Moreover, on both 6 and 10 days, ferric reduction activities were higher in RNAi ovules than in Null and overexpressors under Fe-deficient condition. In addition, in RNAi line, ferric reduction activities were also higher in Fe-deficient ovules than in Fe-sufficient ovules (**Figures 5I,J**). It indicates that cytosolic *APX* suppression increased ferric reduction activity to enhance the tolerance of ovules to Fe deficiency. Furthermore, the cytosolic *APX* suppressed seedlings were also showed the enhance tolerance to Fe deficiency. When the seedlings of overexpressing, control (Null and WT), and RNAi lines (IAO) were cultured in 1/2 Hoagland nutrient solution without iron for 2 weeks, the leaves of the RNAi lines (IAO) remained green while the leaves of overexpressors and controls exhibited chlorosis (Supplementary Figure 3). Therefore, the suppression of cytosolic *APX* in cotton leads to reduced symptoms of Fe deficiency.

### ASC Enhances Tolerance to Iron Deficiency

**FIGURE 6:**
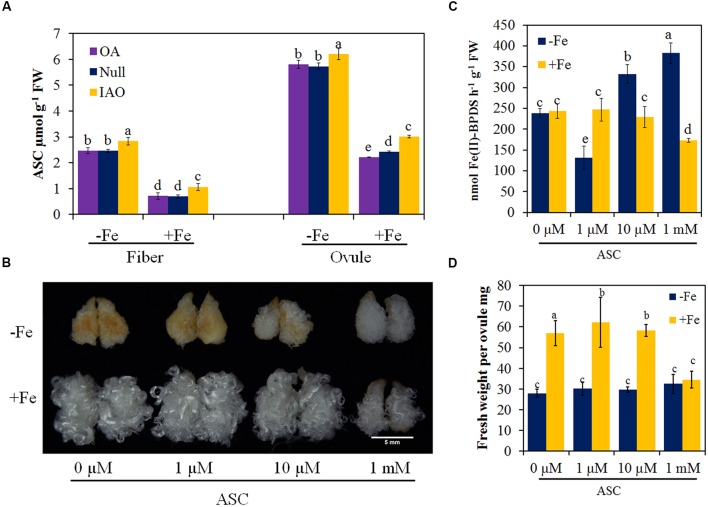
**Optimal ascorbate (ASC) increases the tolerance of ovules to Fe deficiency. (A)** The concentrations of ASC in ovules and fibers of *APX* transgenic lines and the control cultured in Fe-deficient (-Fe) or Fe-sufficient medium (+Fe) after 10 days culture. -Fe, 0 μM Fe; +Fe, 60 μM Fe. The phenotype **(B)**, Ferric reduction activities **(C)** and fresh weight per ovule **(D)** of ovules cultured in Fe deficient (-Fe) or Fe sufficient (+Fe) medium applied with four levels of ASC. Scale bar: 5 mm **(B)**. Error bars represent SD. Every sample includes four biological replicates. Values with different letters above bars in the histogram indicate significant differences (one-way ANOVA and Duncan’s multiple comparisons, *P* < 0.05).

To find why the cytosolic *APX* suppression lines showed higher tolerance to Fe deficiency, we measured the contents of ASC in ovules and fibers after 10 days culture in Fe-deficient and -sufficient conditions. Fe deficiency induced the increase of the ASC contents in OA and Null ovules and fibers (**Figure 6A**). The increase of ASC content in ovules and fibers in the RNAi lines was greater than OA and Null (**Figure 6A**). This is consistent with the possibility that higher contents of ASC might contribute to higher tolerance to Fe deficiency.

To further substantiate a protective role for ASC in Fe-limited conditions, increasing concentrations of ASC (0, 1, 10 μM and 1 mM) were added to the ovule culture medium without Fe or with the standard concentration of Fe. After 10 days culture, the exogenous application of ASC was found to increase the tolerance of ovules to Fe deficiency, and the higher the ASC in Fe deficient medium, the more protection was observed (**Figure 6B**). Ferric reduction activities significantly increased in fiber-bearing ovules cultured in Fe-deficient medium in the presence of exogenous ASC (**Figure 6C**). Although the fresh weight per ovule did not increase, ASC protected against Fe-deficiency induced browning of ovules and fibers (**Figure 6D**). However, application of ASC to cultures in Fe-sufficient medium suppressed the ovule and fiber development, and ferric reduction activities also decreased (**Figures 6B,C**), and the higher the ASC concentration, the lower the ferric reduction activities (**Figures 6B,D**).

To investigate why the RNAi lines improves tolerance to Fe deficiency, the expression levels of marker genes of the response to Fe uptake were analyzed by qRT-PCR. *FRO2/7*, *IRT1/2/3*, *FIT1*, and *bHLH* transcription factors were found to be upregulated in ovules of RNAi lines compared with the control under Fe deficient condition, with particularly large increases for *FRO2*, *IRT1*, *ILR3*, and *bHLH92* (Supplementary Figure 4). Therefore, the upregulation of Fe uptake genes in cytosolic *APX* suppression lines is associated with the improved tolerance to Fe deficiency. These results show that ASC can improve the tolerance of ovules to Fe deficiency by activating the upregulation of Fe uptake genes.

### ASC Maintains ABA Levels in RNAi Lines

**FIGURE 7:**
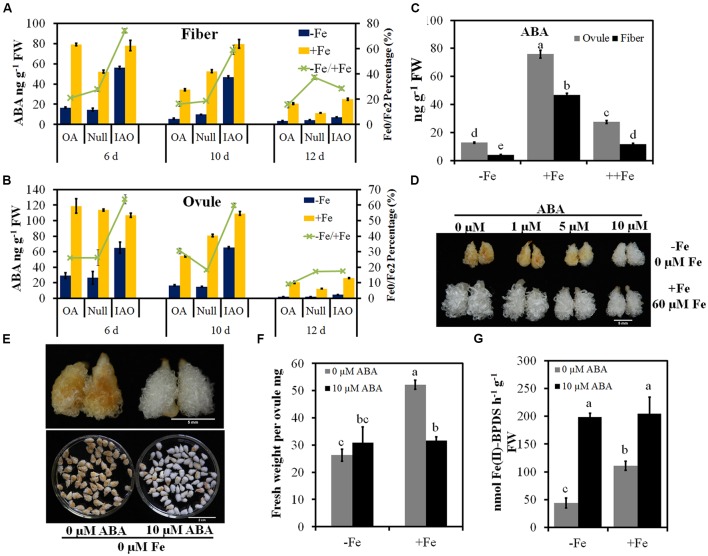
**Reduced decrease in ABA content in cytosolic *APX* RNAi (IAO) tissues improves tolerance to Fe deficiency stress**. Quantification of ABA concentrations in fibers **(A)** and ovules **(B)** of *APX* transgenic lines and the control after 6, 10, or 12 days culture. -Fe, 0 μM Fe; +Fe, 60 μM Fe; ++Fe, 240 μM Fe. OA, *GhAPX1* overexpression line; Null, the control; IAO, cytosolic *APXs* suppression line. **(C)** Concentrations of ABA in ovules and fibers cultured with three concentrations of Fe for 10 days. Each treatment includes four biological replicates. Error bars represent SD. Values with different letters above bars in the histogram indicate significant differences (one-way ANOVA and Duncan’s multiple comparisons, *P* < 0.05). **(D,E)** a serious of ABA application suppresses symptoms induced by Fe deficiency after 10 days culture. Scale bars: 5 mm **(D)**. **(F)** The exogenous application of 10 μM ABA could not restore the fresh weight per ovule inhibited by Fe deficiency. **(G)** The exogenous application of 10 μM ABA completely recover the ferric reduction of ovules treated with Fe deficiency.

To understand why an increase of ASC in RNAi lines improves tolerance to Fe deficiency, we assayed endogenous hormones of transgenic and control ovules and fibers treated for 6, 10, or 12 days under Fe-deficient and -sufficient conditions. The changes of IAA, JA, or JA-ILE were not consistent with phenotypes of transgenic and control ovules and fibers, and the exogenous application of JA could not alter the Fe deficiency induced browning (Supplementary Figure 5). Therefore, we focused on the effect of ABA in the resistance to the Fe deficiency. For the levels of ABA in ovules and fibers of wild type grown on Fe-deficient medium were significantly reduced compared with those grown on Fe-sufficient medium after 6–12 days culture (**Figures 7A–C**). Although, the contents of ABA in ovules and fibers in RNAi lines decreased under Fe-deficient condition, they were still higher than that in overexpressor and Null line (**Figures 7A,B**). The decrease of ABA contents in RNAi ovules and fiber was also slower than in overexpressor and Null under Fe deficiency treatment for 6 or 10 days (**Figures 7A,B**).

To determine whether exogenous ABA could alleviate the tissue browning induced by Fe deficiency, a series of ABA concentrations (0, 1, 5, and 10 μM) was added to the Fe-deficient medium. After 10 days cultures, the exogenous application of 10 μM ABA significantly inhibited the development of browning, although the development of ovules and fibers was not restored (**Figures 7D–F**). However, the exogenous application of 10 μM ABA could significantly induced the increase of ferric reduction activity under Fe deficient or Fe sufficient condition. Especially under Fe deficiency condition, 10 μM ABA could completely restore the same level of ferric reduction activity as normal condition (**Figure 7G**). These results show that ABA could alleviate symptoms of Fe deficiency.

**FIGURE 8:**
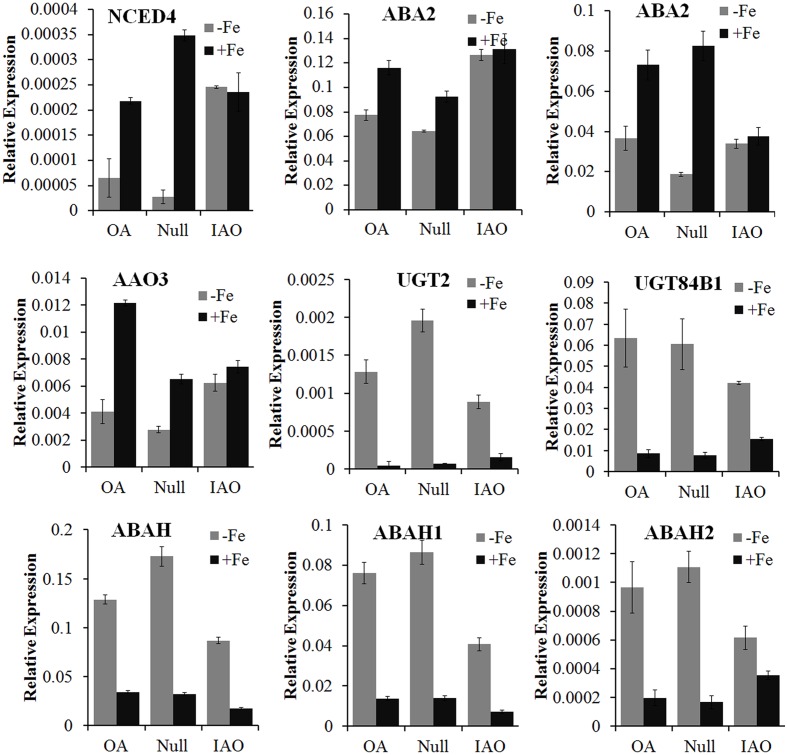
**Reverse transcription polymerase chain reaction (qRT-PCR) analysis of ABA biosynthesis and metabolism genes in ovules of transgenic cotton and control, cultured for 10 days under Fe-deficient or Fe-sufficient conditions**. The relative expression levels of genes were normalized against the expression of the cotton *GhUB7* gene. Error bars represent SD of three technological repeats.

To investigate why the contents of ABA decreased more slowly in RNAi lines compared to overexpressor and Null lines, the expression of genes involved in ABA biosynthesis, signaling and metabolism were analyzed by qRT-PCR. The expression of biosynthesis genes *NCED4*, *ABA2*, and *AAO3* was suppressed by Fe deficiency in overexpressor and Null tissues, but was not downregulated in Fe-deficient ovules of RNAi lines compared to the Fe-sufficient ovules (**Figure 8**). The expression levels of the ABA glycosylation genes *UGT2* and *UGT84B1* and the ABA oxidation gene *ABAH* were induced by Fe deficiency, and the increased rate in RNAi lines was less than in the control (**Figure 8**). Therefore, stable ABA biosynthesis and reduced degradation of ABA in Fe-deficient RNAi ovules would account for the observed slower decrease in ABA content.

To confirm the role of ABA in the Fe-deficiency response, we decided to check whether the decrease of ABA in the ovule could increase the sensitivity to Fe deficiency. With the overexpression of ABA 8′-hydroxylase gene (*GhABAH1*) in cotton, ABA levels were significantly decreased (Supplementary Figures 6A,B). And after 9 days culture, *GhABAH1* overexpressed cotton showed Fe deficiency induced browning, while the control was still white (Supplementary Figure 6C). Therefore, ABA plays a positive role in the resistance to the Fe deficiency induced browning.

## Discussion

### ASC Can Partially Alleviate Symptoms of Fe Deficiency

The cotton ovule culture system described here has proven valuable for improving understanding of metabolic and hormonal interactions under conditions of Fe deficiency. Lack of Fe suppresses the *in vitro* development of ovules and fibers, and after 10 days culture, tissues undergo browning (**Figure 2A**), due to a loss of redox regulation leading to phenolic accumulation and oxidative polymerization (Jin et al., 2007; Rodriguez-Celma et al., 2013). To adapt to nutrient deficiency stress, plants use a range of strategies to cope with the Fe deficiency, and ASC accumulation is one of them. Fe deficiency induces the accumulation of ASC in sugar beet roots and *Arabidopsis* (Zaharieva and Abadia, 2003; Ramirez et al., 2013), and the exogenous application of 1 mM ASC prevents Fe deficiency-induced chlorosis and restores the level of chlorophyll in Fe-deficient *Arabidopsis* seedlings (Ramirez et al., 2013). In pea and *Arabidopsis*, plants employing Strategy I, the outflow of ASC in embryos increased ferric reduction activity that facilitated iron uptake. Mutations in the ASC biosynthesis genes *VTC2-4*, *VTC5-1*, or *VTC5-2* leads to a significant decrease of ferric reduction activity and total iron content in whole seeds (Grillet et al., 2014). All of these results point the critical role of ASC in cell responses to Fe deficiency stress.

Our results also demonstrate that ASC improves the tolerance of cotton ovules and fibers to Fe deficiency. Firstly, Fe deficiency induces an increase of ASC in tissues, and the *myo*-inositol and galacturonic acid pathway are activated (**Figure 4**). Secondly, the suppression of cytosolic *APX* gene expression and enzyme activity slows down the ASC metabolic activity under Fe-deficiency (Supplementary Figure 2). Thirdly, suppression of cytosolic *APX* increases ASC contents, associated with higher tolerance to Fe deficiency than the control (**Figure 5**). Finally, exogenous application of ASC partially alleviates the symptoms induced by Fe deficiency, associated with increased ferric reduction activity (**Figure 6**). Therefore, it is likely that ASC accumulation is one of strategies to improve the tolerance to Fe deficiency in Strategy I plants.

The high levels of ASC in Fe-deficient tissues of the cytosolic *APX* RNAi lines were associated with a sharp increase of *IRT1* and *FRO2* expression (Supplementary Figure 4). After 6 or 10 days culture, ferric reduction activity in these lines was also higher under Fe-deficient than -sufficient conditions (**Figures 5I,J**). Exogenous application of ASC to the Fe-deficient medium inhibited the development of stress symptoms and increased ferric reduction activity. IRT1 is essential for Fe uptake and plant growth (Vert et al., 2002; Zelazny and Vert, 2015). FRO2 belongs to a superfamily of favocytochromes that transport electrons across membranes and has ferric chelate reductase activity (Robinson et al., 1999). In *Arabidopsis*, overexpression of *AtFRO2* increases ferric chelate reductase activity under the condition of Fe deficiency, which confers tolerance to growth in low Fe (Connolly et al., 2003). We therefore propose that ASC increases the tolerance to Fe deficiency by activating the absorption of Fe.

### ABA Increases Tolerance to Fe Deficiency

Abscisic acid regulates many developmental processes and adaptive stress responses in plants (Cutler et al., 2010), and it has been demonstrated to be involved in the Fe deficiency response. Fe deficiency rapidly induced the accumulation of ABA in roots as an early stage response, followed by a decrease in ABA concentrations. Exogenous application of 0.5 μM ABA alleviates Fe deficiency-induced chlorosis (Lei et al., 2014). The *Arabidopsis cpl1-2* (*RNA polymerase II C-terminal domain-phosphatase-like1*) mutant shows improved tolerance to Fe deficiency through cross talk between Fe deficiency signaling and the osmotic stress or ABA signaling pathways (Aksoy et al., 2013). Moreover, ABA up-regulates Fe long-distance transport genes, such as *AtFRD3*, *AtYSL2* and *AtNAS1*, to increase the reutilization and transport of Fe from root to shoot under Fe deficiency (Lei et al., 2014).

In *Arabidopsis*, ferric chelate reductase activity was associated with tolerance to Fe deficiency, and overexpression of *AtFRO2* increases ferric chelate reductase activity in root, which confers tolerance to growth in low Fe (Connolly et al., 2003). According to our ovule culture results, the exogenous application of ABA suppressed the development of browning induced by Fe deficiency, which associated with the complete recovery of ferric reduction activity (**Figure 7**). **Figure 1** also indicated that the change of ferric reduction activity of ovules was coupled with the development of browning. In 6 days, the high ferric reduction activity was along with white ovules and fibers under Fe deficiency. When the ferric reduction activity was significantly decreased in 10 days, the browning was occurred (**Figure 1**). However, the exogenous application of ABA could not upregulated the expression levels of marker genes involved in Fe uptake under Fe deficient or sufficient conditions (Supplementary Figure 7). Therefore, ABA may play a positive role in Fe deficiency adaption by maintain the ferric reduction activity.

### ASC Maintained the Contents of ABA to Suppress the Browning Induced by Fe Deficiency

As indicated above, ASC is exported to modulate ferric reduction to promote Fe uptake in pea and *Arabidopsis* embryos (Grillet et al., 2014). It also suggested that ASC may act as an anti-oxidative molecule to increase tolerance to Fe deficiency, through its ROS scavenging ability (Ramirez et al., 2013). Here, we propose that ASC improves tolerance to Fe deficiency in cytosolic *APX* RNAi tissues through maintaining ABA concentrations (**Figures 6A** and **7**). We found that after 6–12 days culture, Fe deficiency induces a significant decrease of ABA, possibly due to the suppression of ABA biosynthesis or ABA inactivation or degradation (**Figure 8**). Exogenous application of 10 μM ABA was found to inhibit Fe-deficiency symptoms. However, the reduction of ABA in cytosolic *APX* RNAi tissues was significantly slower than in overexpressors or the control (**Figures 7A,B**). The decrease of ABA level in cotton increases the sensitivity to Fe deficiency (Supplementary Figure 6). It is consistent with the possibility that ASC improves tolerance to Fe deficiency through regulation of ABA levels.

An important question is how ASC controls the ABA concentration under Fe-deficient condition. In addition to its anti-oxidative properties, ASC also acts as a cofactor of many enzymes, such as the Fe^2+^ and 2OG-dependent dioxygenases family of enzymes, including Jumonji C-domain-containing histone demethylases or ten-eleven translocation (TET) dioxygenases (Foyer and Noctor, 2011; Minor et al., 2013; Monfort and Wutz, 2013; Young et al., 2015). The Jumonji C (JmjC)-domain-containing histone demethylase can demethylate mono-, di-, and trimethylated histone lysine residues *in vitro* in a mechanism dependent on ASC (Klose et al., 2006; Tsukada et al., 2006). It has been reported that ascorbate regulates the H3K9 methylation status of the core pluripotency loci to control the conversation of iPSC cell (Kooistra and Helin, 2012; Accari and Fisher, 2015; Camarena and Wang, 2016).

Histone methylation plays an essential role in diverse biological processes such as flowering, plant development, and stress responses, among others (Liu et al., 2010). It has now also demonstrated that histone methylation is involved in the Fe deficiency response. In *Arabidopsis*, Fe deficiency causes the disassociation of SKB1 from chromatin and leads to a decrease in the level of H4R3sme2, thereby enhancing Fe uptake (Yue et al., 2013; Fan et al., 2014). Histone acetyltransferase GCN5 impairs Fe transport from root to shoot by positively controlling the expression of *FRD3* via H3K9/14ac modifications (Xing et al., 2015). Several studies have revealed that FRD3, a citrate eﬄux transporter, releases citrate into the root xylem to form a ferric-citrate complex that is subsequently transported from root to shoot (Rogers and Guerinot, 2002; Green and Rogers, 2004; Roschzttardtz et al., 2011). We therefore speculate that the stable ASC in cytosolic *APX* RNAi lines, under Fe deficient conditions, might change the state of histone methylation of genes related to ABA biosynthesis, metabolism or signaling transduction to cope with the Fe deficiency stress. Future studies will be required to confirm this.

## Conclusion

A long time of Fe deficiency significantly suppressed the accumulation of ABA and led to the decrease of ferric reduction activity, which resulted in the browning of ovule and fibers. Meanwhile, Fe deficiency inhibited cytosolic APX activity to increase the content of ascorbate in cells. Accumulated ascrobate upregulated Fe uptake related genes and activated ferric reduction activity one side, on the other side it also slowed down the decrease of ABA level in Fe deficiency treated ovules by activating the ABA biosynthesis genes and suppressing the ABA degradation genes. The maintainable ABA in Fe deficiency ovule could inhibit the development of ovule browning. Therefore, it may be a feedback regulation pathway that Fe deficiency-induced the increase of ascorbate by the suppression of APX to slow down the decrease of ABA (Supplementary Figure 8). The feedback regulation way together with the effect of ascorbate in Fe uptake increases the tolerance of ovule to the Fe deficiency.

## Author Contributions

LT and XZ conceived the original research plans; KG, LT, and XZ designed the experiments; KG performed most of the experiments and analyzed the data; PW and XD provided assistance in the experiments; SY and ML constructed the *GhABAH1* overexpressed cotton; KG wrote the article with contributions of all the authors; LT and XZ supervised and complemented the writing.

## Conflict of Interest Statement

The authors declare that the research was conducted in the absence of any commercial or financial relationships that could be construed as a potential conflict of interest.
