# Supplementary material for: Ascorbate Alleviates Fe Deficiency-Induced Stress in Cotton (Gossypium hirsutum) by Modulating ABA Levels
Source: Front Plant Sci. 2017 Jan 4;7:1997. doi: 10.3389/fpls.2016.01997 (PMC5209387; doi:10.3389/fpls.2016.01997)
Supplement: Supplementary file 2 [file Data_Sheet_1.docx]

Supplementary Material

**Ascorbate alleviates Fe deficiency-induced stress in cotton (*Gossypium hirsutum*) by modulating ABA levels**

**Authors:** Kai Guo, Lili Tu*, Pengcheng Wang, Xueqiong Du, Shue Ye, Ming Luo and Xianlong Zhang

* **Correspondence Author:** Lili Tu, National Key Laboratory of Crop Genetic Improvement, Huazhong Agricultural University, Wuhan 430070, Hubei, China. Tel: +86 027 87283955; E-mail: [lilitu@mail.hzau.edu.cn](mailto:lilitu@mail.hzau.edu.cn).


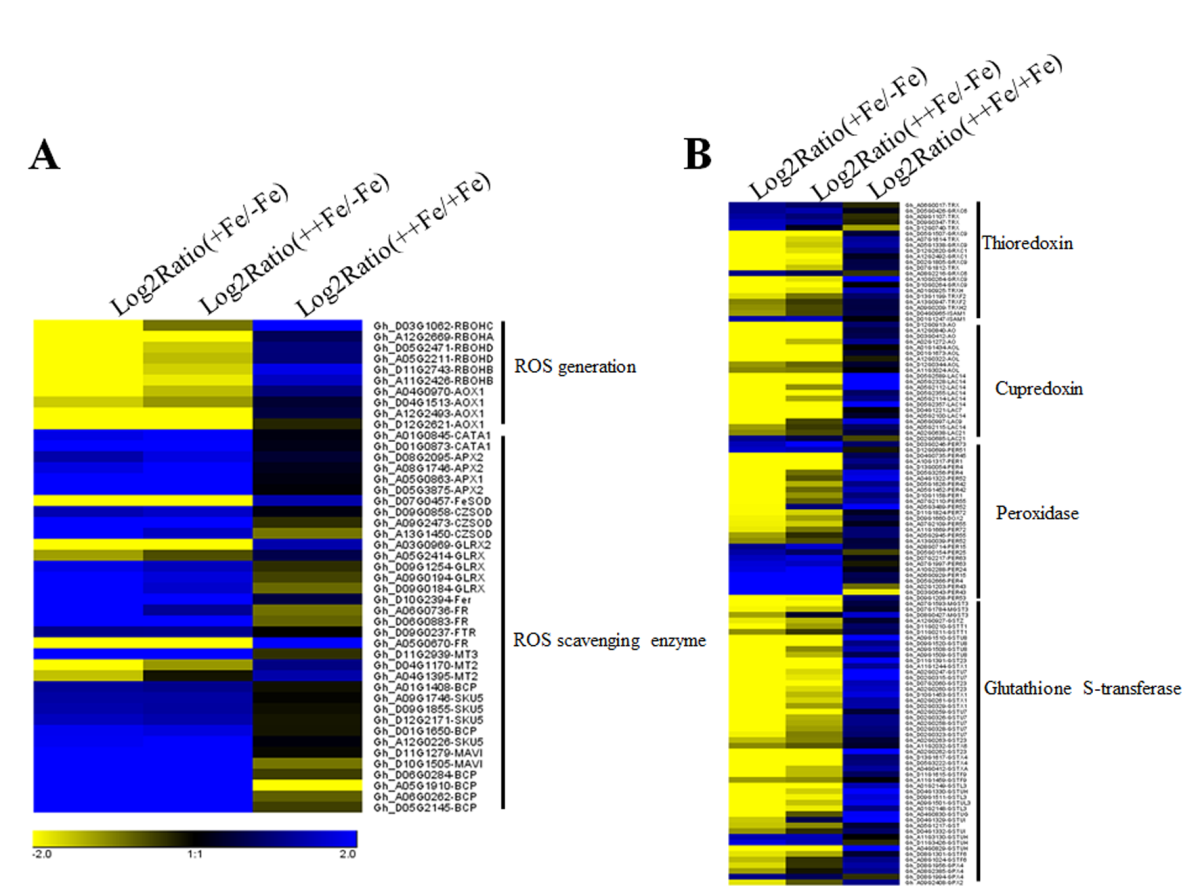


**Supplementary Figure 1.** **Cluster of ROS generation and scavenging genes in 10 d fibers treated with three concentrations of Fe.** -Fe, 0 µM Fe; +Fe, 60 µM Fe; +Fe, 240 µM Fe. **(A)** Response of ROS generation and scavenging genes to the level of Fe in medium. **(B)** Response of genes encoding the enzymes thioredoxin, cupredoxin, peroxidase and glutathione S-transferase to different concentrations of Fe.


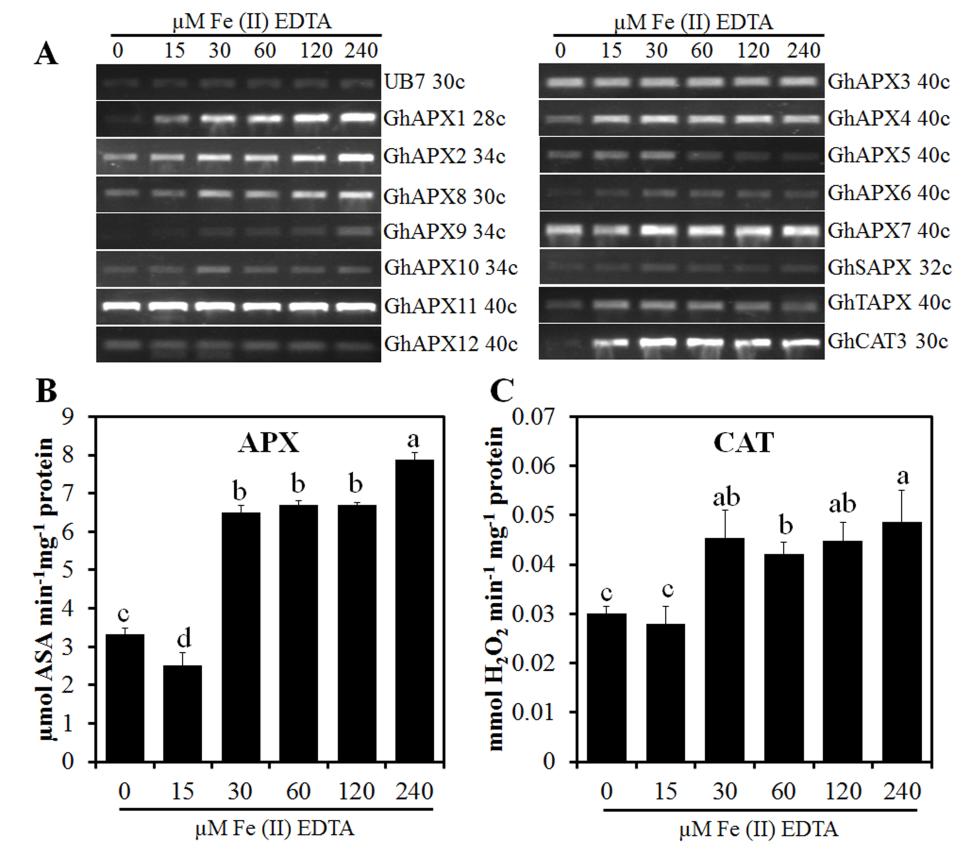


**Supplementary Figure 2.** **Different concentrations of Fe affect transcript levels and enzyme activities of APX and CAT. (A)** RT-PCR analysis of *APX* and *CAT* genes in ovules treated for 10 d under different concentrations of Fe. **(B and C)** Enzyme activities for APX **(B)** and CAT **(C)** in ovules treated with six concentrations of Fe for 10 d. Error bars represent SD. Results are based on six replicates. Values with different letters above bars in the histogram indicate significant differences (one-way ANOVA and Duncan’s multiple comparisons, *P* < 0.05).


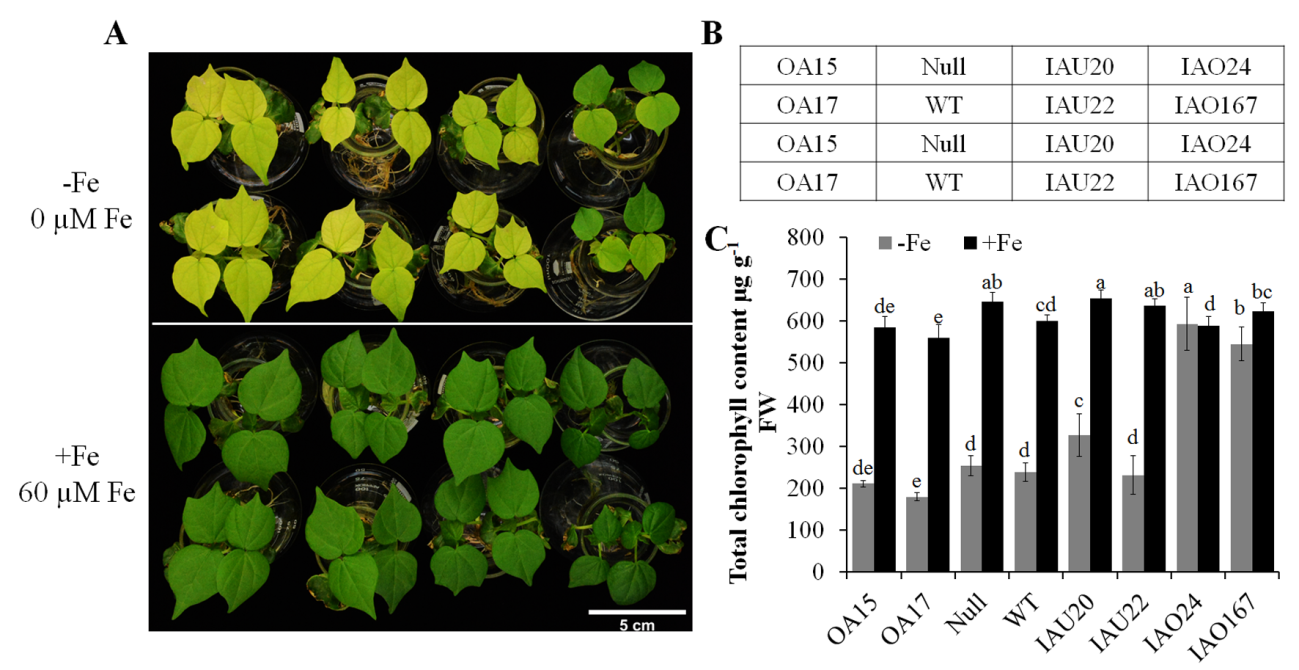


**Supplementary Figure 3. Seedlings of cytosolic *APX* suppressed line (IAO) show insensitivity to Fe deficiency. (A)** Phenotype of seedlings for *APX* transgenic cottons and the controls cultured for two weeks in 1/2 Hoagland medium with or without Fe. Before seedlings were transferred into the 1/2 Hoagland medium without Fe, seeds of 8 lines were germinated and grown in 1/2 MS medium for one week without iron. **(B)** The arrangement of transgenic seedlings in panel **A**. **(C)** Total chlorophyll content in first leaf of seedlings treated with 0 µM Fe or 60 µM Fe (mean ± sd, n=6). Values with different letters above bars in the histogram indicate significant differences between different line in -Fe or +Fe (one-way ANOVA and Duncan’s multiple comparisons, *P* < 0.05). OA15 and OA17, *GhAPX1* overexpressing lines; Null, the negative control; WT, wild type cotton YZ1; IAU20 and IAU22, *GhAPX1* suppressed lines; IAO24 and IAO167, cytosolic *APX* suppressed lines.


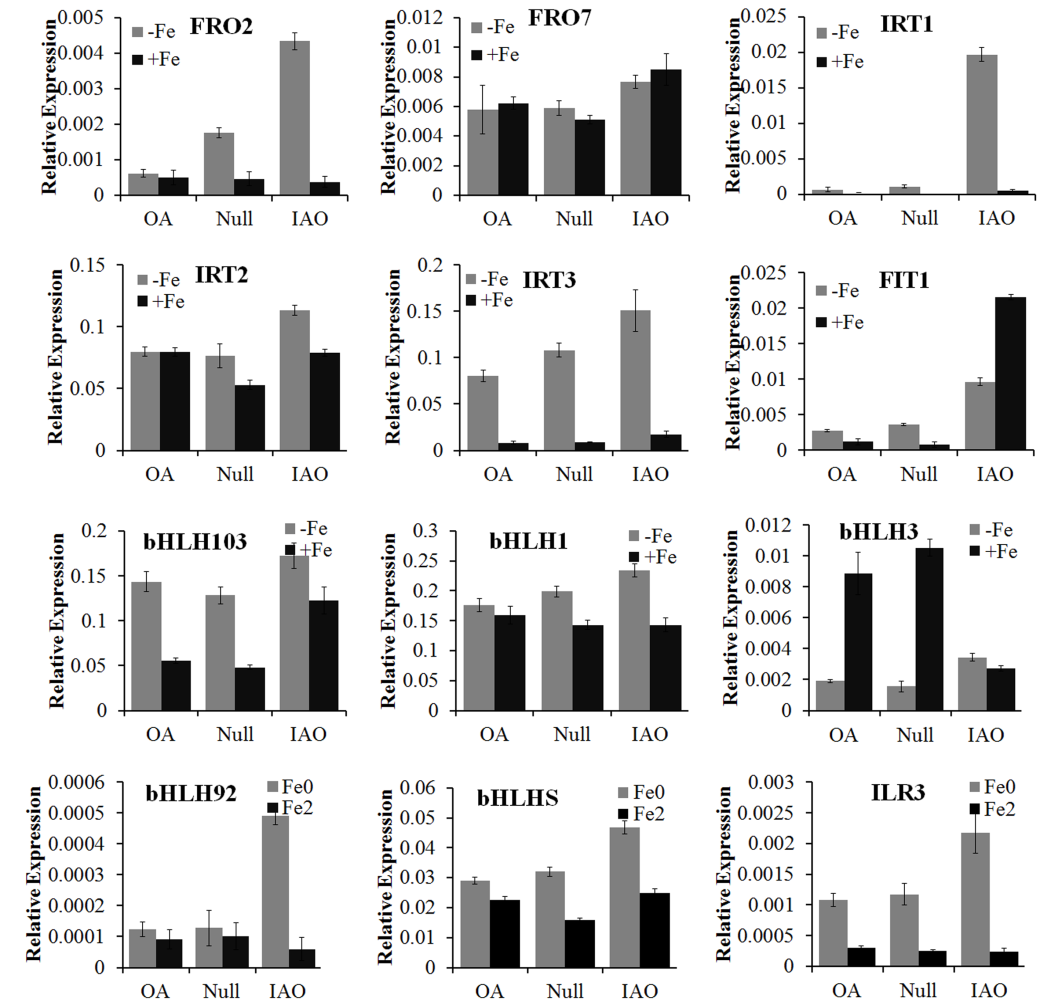


**Supplementary Figure 4.** **qRT-PCR analysis of Fe uptake genes in ovules of transgenic cotton and controls cultured for 10 d under Fe-deficient or -sufficient conditions.** -Fe, 0 µM Fe; +Fe, 60 µM Fe. Relative expression levels of genes were normalized against the expression of the cotton *GhUB7* gene. Error bars represent SD of three technological repeats. OA, *GhAPX1* overexpression line; Null, the control; IAO, cytosolic *APXs* suppression line.

**
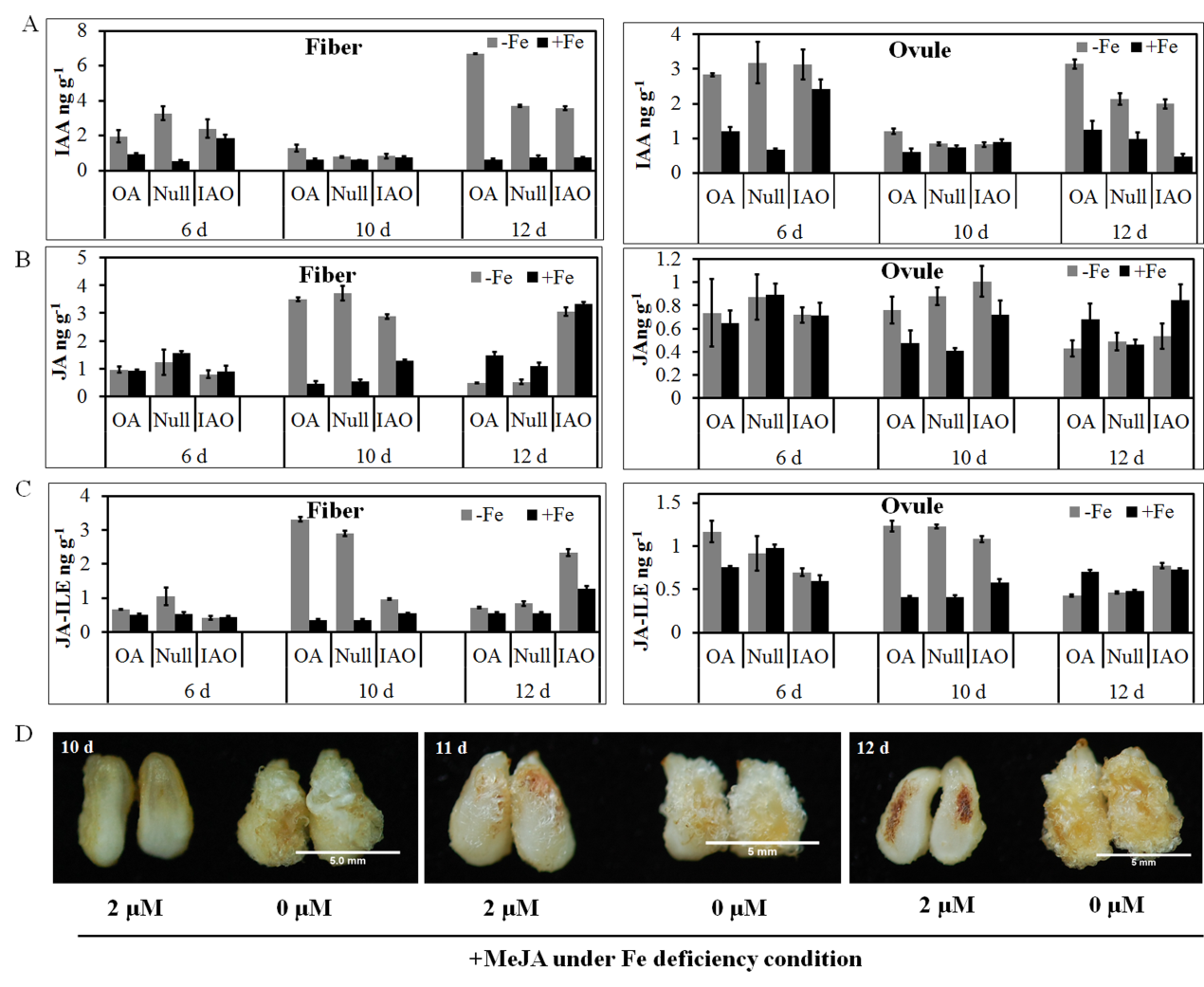
Supplementary Figure 5.** **Quantification of hormones in fibers and ovules of *APX* transgenic lines and the control after 6, 10 or 12 d culture.** -Fe, 0 µM Fe; +Fe, 60 µM Fe; OA, *GhAPX1* overexpression line; Null, the control; IAO, cytosolic *APXs* suppression line. Each treatment includes four biological replicates. Error bars represent sd. **(A)** The content of IAA in different lines. **(B)** The content of JA in different lines. **(C)** The content of JA-ILE in different lines. **(D)** Phenotypes of Fe-deficient ovules treated with 2 µM MeJA for 10, 11 and 12 d.


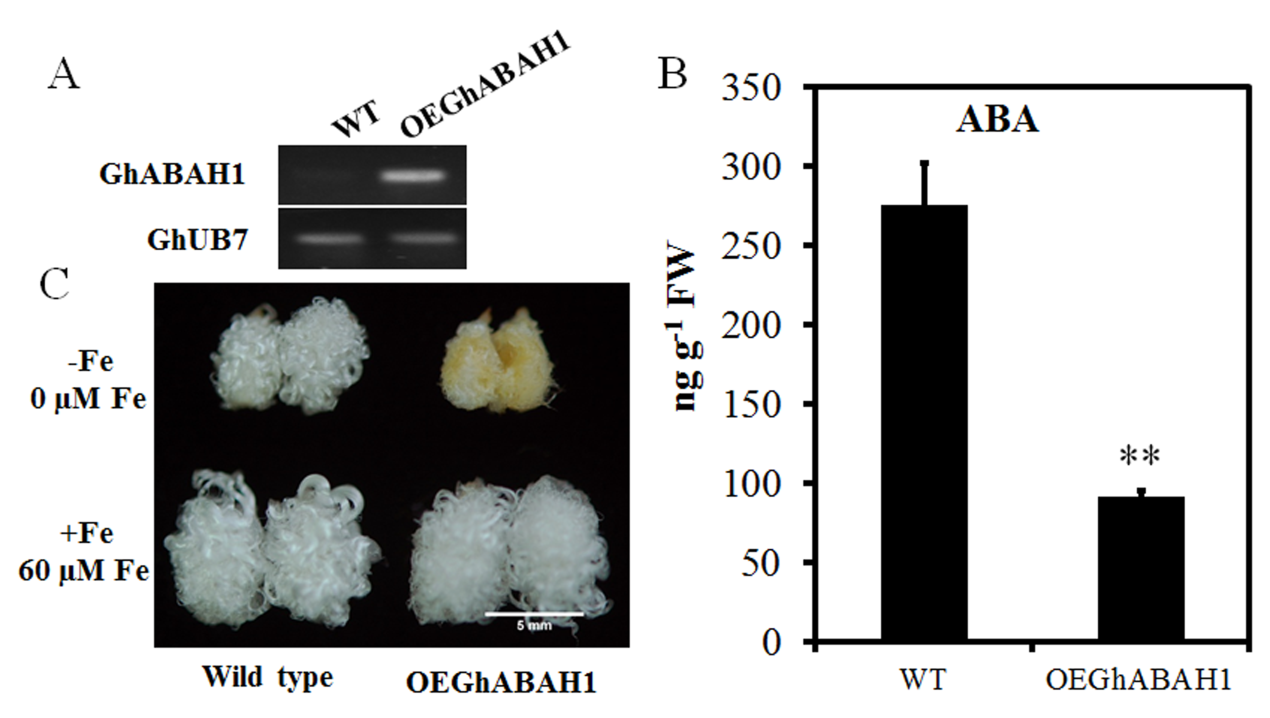


**Supplementary Figure 6.** **The sensitivity analysis of cotton ABA 8'-hydroxylase (*GhABAH1*) overexpressed cotton to Fe deficiency. (A)** The expression level detection of *GhABAH1* in leaf of wild type and overexpressor by RT-PCR. **(B)** The contents of ABA in leaf of wild type and overexpressor. (mean ± sd, n=4) ** above bars in the histogram indicate significant differences (Student’s *t* test; **, *p* < 0.01). **(C)** he phenotypes of *GhABAH1* overexpressor (OEGhABAH1) and wild type fiber-bearing ovules treated for 9 d under Fe-deficient and sufficient Fe condition. Scale bar: 5 mm.


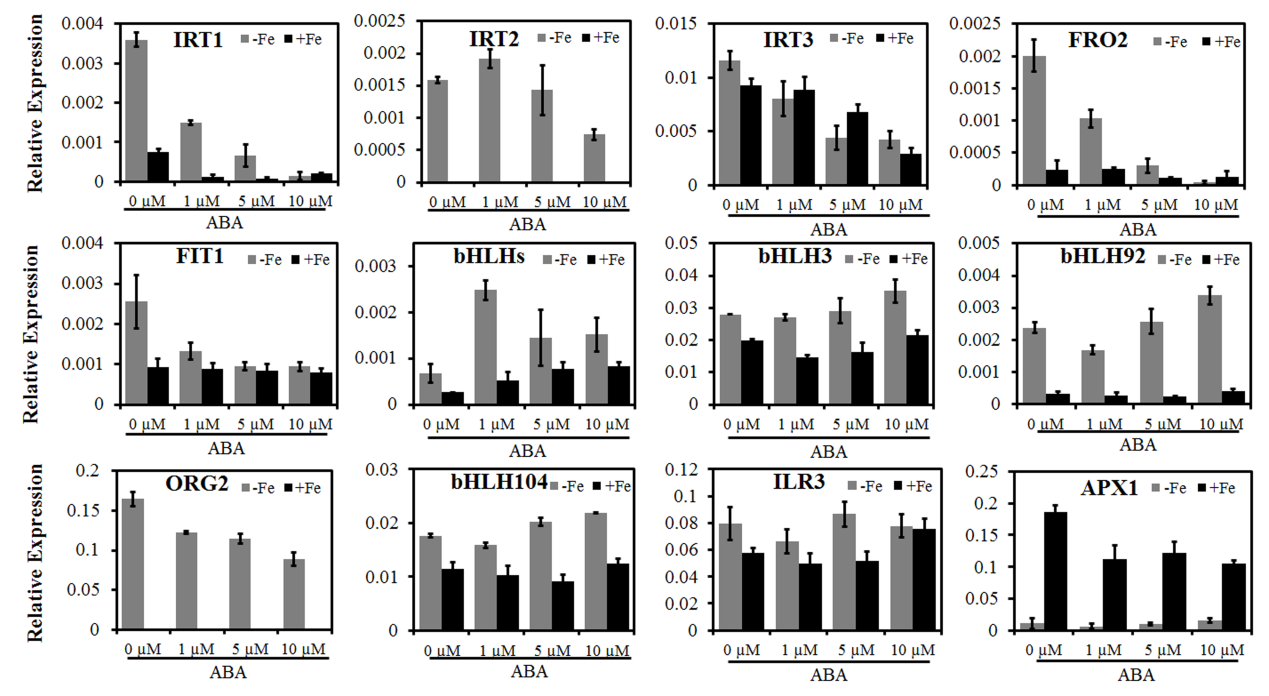


**Supplementary Figure 7. The expression levels of Fe uptake related genes in ovules treated with a serious of ABA under Fe deficient and sufficient Fe conditions by qRT-PCR.**

**
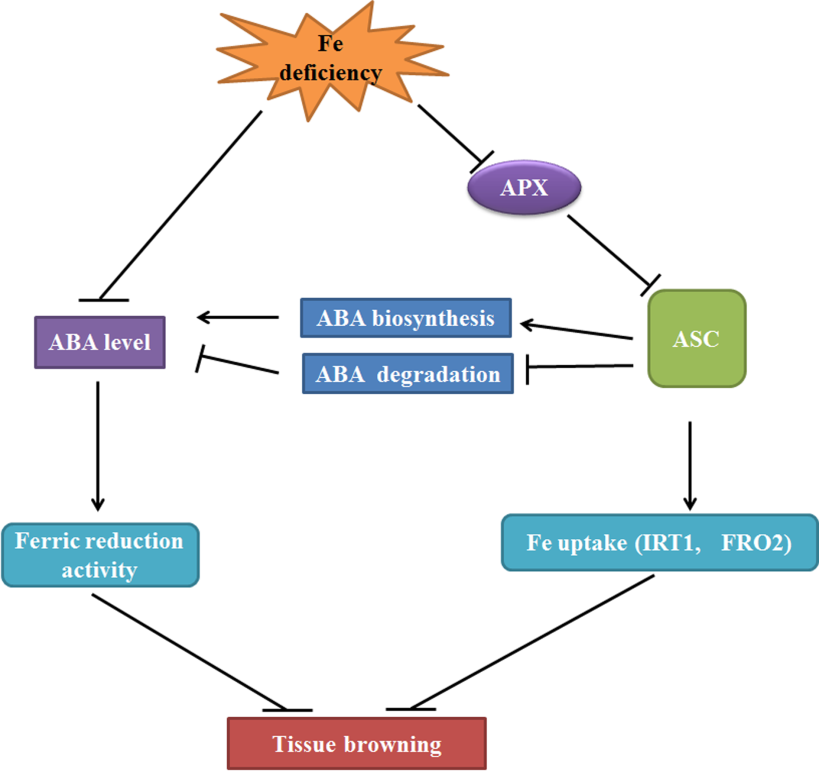
**

**Supplementary Figure 8. Schematic model of ascorbate and ABA in response to Fe deficiency in cotton ovules.**
